# Comprehensive assessment of emergency departments in county-level public hospitals: a multicenter descriptive cross-sectional study in Henan province, China

**DOI:** 10.3389/fpubh.2023.1301030

**Published:** 2023-11-14

**Authors:** Yanwei Cheng, Xue Cao, Jiange Zhang, Lijun Xu, Lijie Qin

**Affiliations:** ^1^Department of Emergency, Henan Provincial People’s Hospital, People’s Hospital of Zhengzhou University, People’s Hospital of Henan University, Zhengzhou, China; ^2^Department of Rheumatology and Immunology, Henan Provincial People’s Hospital, People’s Hospital of Zhengzhou University, People’s Hospital of Henan University, Zhengzhou, China

**Keywords:** emergency department, county-level hospitals, public hospitals, healthcare disparities, China

## Abstract

**Background:**

Emergency Departments (EDs) play a crucial role in providing immediate medical care, particularly in densely populated countries like China. While previous research has predominantly focused on well-funded urban hospitals, this study offers a comprehensive evaluation of EDs in county-level public hospitals in Henan province, China, aiming to identify disparities and challenges.

**Methods:**

A descriptive cross-sectional survey was conducted in 382 public hospitals across Henan province, China, from July 1, 2023, to August 1, 2023. Data were collected using an electronic questionnaire covering hospital information, human resources, infrastructure, clinical capabilities, and operational capacities. The data collection period for this survey spanned from January 1 to December 31, 2022.

**Results:**

With a remarkable 94.0% response rate, our study reveals significant disparities in county-level public hospitals compared to their provincial or municipal counterparts in Henan Province, China. County-level hospitals, which constitute 266 of the total 342 surveyed facilities, exhibit notable differences, including fewer doctors (median: 11 vs. 23, *p* < 0.0001) and nurses (median: 18 vs. 37, *p* < 0.0001). Additionally, a higher proportion of junior doctors is observed in these hospitals, while senior medical staff are more prevalent in provincial or municipal hospitals (*p* < 0.001). County-level hospitals also face resource challenges, with fewer beds in the emergency room (median: 4 vs. 7, *p* = 0.0003) and limited proficiency in advanced clinical procedures such as POCT, fiberoptic bronchoscopy, CRRT, ECMO, ultrasound equipment operation, and intraosseous infusion, with significant differences noted in most of these capabilities (*p* < 0.05). Operational capabilities show distinctions as well, with county-level hospitals managing a lower patient volume (median: 14,516 vs. 34,703, *p* < 0.0001) and handling fewer pre-hospital CPR cases (median: 33 vs. 89, *p* < 0.0001). In-hospital CPR success rates are also lower in county-level hospitals (median ROSC: 25.0% vs. 42.8%, *p* = 0.0068).

**Conclusion:**

While provincial or municipal hospitals enjoy better resources, county-level hospitals, especially crucial in less urbanized regions, face substantial challenges. Addressing these disparities is imperative, necessitating targeted investments, improved infrastructure, enhanced clinical training, and the adoption of innovations like telemedicine to enhance the quality of emergency care.

## Introduction

Emergency Departments (EDs) serve as crucial entry point for immediate and often life-saving medical care. They are essential components of a healthcare system, providing 24-h services for patients with various conditions, ranging from acute medical emergencies to less urgent issues (1). In China, with its vast population exceeding 1.4 billion, EDs take on an even more critical role (2). Therefore, establishing a comprehensive and highly efficient framework for EDs becomes imperative.

In China, the healthcare infrastructure is categorized into primary, secondary, and tertiary levels (3). Primary care is provided by community health centers, offering essential medical services (4). Provincial or municipal hospitals, on the other hand, offer advanced tertiary care (5). County-level public hospitals, situated between these levels, serve as essential secondary healthcare hubs. These hospitals play a particularly crucial role in less urbanized regions, where they frequently grapple with challenges like limited resources, staffing constraints, and a high patient volume (6). Unlike their counterparts in major cities, which enjoy easier access to specialized facilities, county-level public hospitals often shoulder a substantial healthcare burden.

The rapid urbanization and aging population in China have led to an upsurge in medical emergencies, spanning from traumatic injuries to exacerbations of chronic diseases (7). These trends exert immense pressure on EDs, frequently resulting in overcrowding, prolonged wait times, and potential compromises in patient care. Given the high stakes associated with emergency care, it is paramount to evaluate the current state of EDs in county-level hospitals. This evaluation will help identify gaps, challenges, and opportunities for improvement.

While several studies have explored ED care in tertiary hospitals in major Chinese cities (8, 9). existing research disproportionately focuses on well-funded and well-staffed urban hospitals. This leaves a substantial knowledge gap concerning county-level public hospitals. Therefore, this study seeks to address this gap by providing a comprehensive evaluation of the state of EDs in county-level public hospitals in Henan province, China. The insights gained from this research will serve as a vital resource for policymakers, hospital administrators, and healthcare providers in identifying shortcomings and devising strategies to enhance emergency care services. This is especially relevant as the Chinese government actively pursues healthcare reform, including initiatives aimed at strengthening secondary and tertiary healthcare services.

## Methods

### Study design

This study is a descriptive cross-sectional survey conducted in the EDs of 382 public hospitals located in Henan Province, China. The survey was carried out from July 1, 2023, to August 1, 2023, and included hospitals with more than 500 beds distributed across 85 counties and 17 cities within the province.

### Data collection

Data for this study were collected using an electronic questionnaire administered through the Questionnaire Star software (10), accessible via the smartphone-based platform WeChat. Heads of the EDs designated specific individuals responsible for compiling the relevant data. Data for the year 2022, spanning from January 1 to December 31, were collected, verified by department heads, and subsequently submitted through the Questionnaire Star platform. Completed questionnaires were automatically collected and exported to an Excel file for comprehensive integrity and validity checks.

### Questionnaire

The questionnaire comprised five sections, as detailed in Supplementary material S1. The initial section contained essential information about the hospital and the respondent, including the hospital’s name, location (city or county), and the presence of an independent ED. The second section focused on gathering information about human resources within the ED, including the total number of medical and nursing staff, their demographic profiles, educational qualifications, and professional titles held by doctors and nurses. This section also sought information about the staffing levels of doctors and nurses on duty in the emergency room at any given time. The third section assessed the infrastructure of the emergency unit, investigating the availability and number of beds in various sections such as the emergency room (ER), emergency observation room, emergency intensive care unit (EICU), and pre-hospital emergency care. The fourth section evaluated the clinical skill capabilities available in the ED, including the capacity for point-of-care testing (POCT), independent endotracheal intubation, independent fiberoptic bronchoscopy, autonomous continuous renal replacement therapy (CRRT), utilization of bedside ultrasound equipment, independent extracorporeal membrane oxygenation (ECMO) techniques, and intraosseous (IO) infusion techniques. The fifth section collected data on the basic operational capabilities of the emergency room, including the number of patients treated annually, categorized by severity levels based on the expert consensus on emergency pre-examination and triage (11): Level 1 for critical patients, Level 2 for severe patients, Level 3 for urgent patients, and Level 4 for non-urgent patients. This section also recorded annual patient mortality rates and details related to cardiopulmonary resuscitation (CPR) cases, both in pre-hospital and in-hospital settings, including the rate of successful return of spontaneous circulation (ROSC).

### Data analysis

Continuous variables were presented as either mean ± standard deviation (SD) or as median with interquartile range (IQR), contingent upon data normality assessed using the Kolmogorov–Smirnov test. Categorical variables were expressed as counts and proportions. Statistical analysis employed either Student’s t-test or the Mann–Whitney U test for continuous variables, while categorical variables were assessed using either the chi-square test or Fisher’s exact test. All statistical analyzes were performed using GraphPad Prism 9.0 software (GraphPad Software Inc., San Diego, California), with a significance level set at *p* < 0.05.

## Results

### Questionnaire distribution and collection

Out of the 382 questionnaires distributed, 364 were returned, resulting in a response rate of 95.3%. After excluding 22 submissions with incomplete or inaccurate data, the study proceeded with 342 validated questionnaires, achieving an effective response rate of 94.0%.

### Human resources in emergency departments

Table 1 provides a comprehensive overview of human resources in EDs across county-level public hospitals (*n* = 266) and provincial or municipal public hospitals (*n* = 76). The data shows that county-level hospitals exhibit a lower median number of doctors (11 vs. 23, *p* < 0.0001) and nurses (18 vs. 37, *p* < 0.0001). Furthermore, a slightly smaller proportion of male doctors is observed in county-level hospitals (60% vs. 64%, *p* < 0.0001), and there’s a lower prevalence of staff aged 30–40 with advanced educational qualifications. In terms of professional titles, county-level hospitals have a greater percentage of junior doctors, while provincial or municipal hospitals have a higher representation of senior doctors and nurses (*p* < 0.001 for both). Notably, no significant disparities are found in the number of doctors or nurses on duty in the emergency room between these hospital types (*p* > 0.65).

**Table 1:** Comparative analysis of staffing characteristics in emergency departments between county-level and provincial or municipal public hospitals.

Characteristics	County-level public hospitals (*n* = 266)	Provincial or municipal public hospitals (*n* = 76)	*p* value
*Doctor demographics*			
Total number of doctors, *n*, median (IQR)	11 (7, 18)	23 (13, 30)	<0.0001
Male doctors, %, median (IQR)	60 (51, 67)	64 (60, 71)	<0.0001
Doctors aged 30–40, %, median (IQR)	43 (37, 57)	60 (54, 67)	<0.0001
*Doctor educational background*			
Master’s degree or higher, %, median (IQR)	0 (0, 0)	9 (5, 22)	<0.0001
Bachelor’s degree, %, median (IQR)	45 (29, 60)	71 (55, 87)	<0.0001
Below bachelor’s degree, %, median (IQR)	54 (39, 71)	7 (0, 28)	<0.0001
*Doctor titles*			
Junior, %, median (IQR)	41 (22, 79)	29 (10, 64)	0.043
Intermediate, %, median (IQR)	40 (29, 50)	42 (32, 57)	0.1706
Senior, %, median (IQR)	14 (8, 20)	22 (15, 30)	<0.0001
*Nurse demographics*			
Total number of nurses, *n*, median (IQR)	18 (11, 28)	37 (18, 59)	<0.0001
Female nurses, %, Mean (SD)	74 (9)	78 (8)	0.0247
Nurses aged 30–40, %, Mean (SD)	47 (38, 57)	71 (62, 75)	<0.0001
*Nurse educational background*			
Master’s degree or higher, %, median (IQR)	0 (0, 0)	5 (2, 8)	<0.0001
Bachelor’s degree, %, median (IQR)	17 (11, 25)	54 (44, 65)	<0.0001
Below bachelor’s degree, %, median (IQR)	83 (75, 89)	39 (27, 50)	<0.0001
*Nurse titles*			
Junior nurses, %, median (IQR)	72 (58, 82)	62 (49, 77)	0.0058
Intermediate nurses, %, median (IQR)	25 (17, 38)	31 (22, 47)	0.0088
Senior nurses, %, median (IQR)	0 (0, 4)	2 (0, 4)	0.0146
*Emergency room staff*			
Doctors on duty, *n*, median (IQR)	2 (2, 3)	2 (2, 3)	0.6564
Nurses on duty, *n*, median (IQR)	3 (2, 5)	3 (2, 5)	0.6604

### Configuration of emergency department units and bed availability

Both levels of hospitals have a 100% presence of emergency rooms and emergency observation rooms. However, county-level hospitals, in contrast to provincial or municipal hospitals, offer a lower median number of beds in the emergency room (4 vs. 7, *p* = 0.0003) and the emergency observation room (18 vs. 20, *p* = 0.0033) (Table 2). EICUs are more common in provincial or municipal hospitals (83% vs. 59%, *p* = 0.0001), which also feature a higher median number of beds (12 vs. 6, *p* < 0.0001). Pre-hospital emergency care is uniformly operated across both hospital types with a median of 2 ambulances, although the difference is statistically significant (*p* = 0.0332).

**Table 2:** Comparative assessment of facility configuration and bed availability in emergency departments between county-level and provincial or municipal public hospitals.

Characteristics	County-level public hospitals (*n* = 266)	Provincial or municipal public hospitals (*n* = 76)	*p* value
*Emergency room*			
Presence, *n* (%)	266 (100%)	76 (100%)	
Number of beds, *n*, median (IQR)	4 (2, 8)	7 (4, 8)	0.0003
Emergency observation room			
Presence, *n* (%)	266 (100%)	76 (100%)	
Number of beds, *n*, median (IQR)	18 (12, 20)	20 (16, 20)	0.0033
Emergency intensive care unit			
Presence, *n* (%)	157 (59%)	63 (83%)	0.0001
Number of beds, *n*, median (IQR)	6 (0, 12)	12 (8, 12)	< 0.0001
*Pre-hospital emergency care*			
Presence, *n* (%)	266 (100%)	76 (100%)	
Number of ambulances, *n*, median (IQR)	2 (2, 2)	2 (2, 2)	0.0332

### Clinical skill capabilities in emergency departments

Table 3 shows that county-level hospitals exhibit certain disparities in clinical skill capabilities compared to their provincial or municipal counterparts. Specifically, county-level hospitals demonstrated lower proficiency in independently performing POCT (79% vs. 89%) and fiberoptic bronchoscopy (0% vs. 32%). Moreover, county-level hospitals were less adept at independently managing CRRT (9% vs. 29%), ECMO (0% vs. 24%), operating bedside ultrasound equipment (36% vs. 57%), and conducting IO infusion (23% vs. 34%). However, there was no significant difference in their ability to independently execute endotracheal intubation.

**Table 3:** Evaluation of clinical skill capabilities in emergency departments across county-level and provincial or municipal public hospitals.

Characteristics	County-level public hospitals (*n* = 266)	Provincial or municipal public hospitals (*n* = 76)	*p* value
Independently perform point-of-care testing, *n* (%)	211 (79)	68 (89)	0.0441
Independently execute endotracheal intubation, *n* (%)	252 (95)	74 (97)	0.338
Independently perform fiberoptic bronchoscopy, *n* (%)	0 (0)	24 (32)	<0.0001
Independently perform continuous renal replacement therapy, *n* (%)	24 (9)	22 (29)	<0.0001
Independently operate bedside ultrasound equipment, *n* (%)	96 (36)	43 (57)	0.0013
Independently manage extracorporeal membrane oxygenation, *n* (%)	0 (0)	18 (24)	<0.0001
Independently conduct intraosseous infusion, *n* (%)	60 (23)	26 (34)	0.0389

### Basic operational capabilities in emergency rooms

As outlined in Table 4, county-level hospitals manage a lower annual patient volume (median: 14,516 vs. 34,703, *p* < 0.0001) and handle fewer pre-hospital CPR cases (median: 33 vs. 89, *p* < 0.0001) when compared to provincial or municipal hospitals. While the proportions of critical and severe patients are similar in both groups (*p* = 0.321 and *p* = 0.7875, respectively), county-level hospitals handle a higher percentage of non-urgent cases (median: 61.6% vs. 47.9%, *p* = 0.0074). Additionally, provincial or municipal hospitals exhibit higher rates of successful in-hospital CPR (median ROSC: 42.8% vs. 25.0%, *p* = 0.0068).

**Table 4:** Comparative analysis of emergency room basic operational capabilities between county-level and provincial or municipal public hospitals.

Characteristics	County-level public hospitals (*n* = 266)	Provincial or municipal public hospitals (*n* = 76)	*p* value
Total number of patients treated annually, n, median (IQR)	14516 (5,907, 26,803)	34703 (20,029, 63,525)	< 0.0001
Critical patients, %, median (IQR)	0.96 (0.42, 2.83)	0.89 (0.40, 1.38)	0.321
Severe patients, %, median (IQR)	4.1 (1.5, 9.2)	3.6 (2.0, 8.8)	0.7875
Urgent patients, %, median (IQR)	28.4 (14.8, 44.8)	43.5 (20.7, 59.9)	0.0035
Non-urgent patients, %, median (IQR)	61.6 (38.8, 80.4)	47.9 (26.3, 72.8)	0.0074
Annual patient deaths, n, median (IQR)	24 (8, 58)	37 (19, 96)	0.0023
Annual pre-hospital CPR cases, *n*, median (IQR)	33 (16, 77)	89 (39, 148)	< 0.0001
Cases of successful ROSC, %, median (IQR)	11.4 (4.2, 28.0)	14.3 (6.1, 31.2)	0.2495
Annual in-hospital CPR cases, *n*, median (IQR)	25 (9, 61)	36 (17, 124)	0.0029
Cases of successful ROSC, %, median (IQR)	25.0 (14.0, 53.3)	42.8 (23.2, 60.0)	0.0068

## Discussion

The present study provides a comprehensive assessment of EDs in county-level public hospitals in Henan province. It highlights substantial in staffing, infrastructure, clinical skills, and operational capabilities between county-level and provincial or municipal public hospitals, reflecting disparities commonly observed in rural and urban healthcare settings worldwide. For instance, rural hospitals in the United States face challenges similar to county-level hospitals in China, including staffing constraints and resource limitations compared to their urban counterparts (12, 13). Similarly, European nations like Spain (13) and Italy (14) have reported disparities in emergency care resources and outcomes between urban centers and their peripheral regions.

In terms of human resources in EDs, our findings align with Yu et al. (15), who also observed that urban and more centrally located hospitals in China tend to have a larger number of medical staff. Studies from Australia have shown that rural EDs tend to be staffed with junior doctors and face challenges in recruiting and retaining experienced professionals (16). The superior presence of senior doctors in provincial or municipal hospitals resonates with the notion that urban areas provide better professional opportunities. Our study further reveals that doctors and nurses at county-level hospitals typically possess undergraduate degrees or lower qualifications. This observation may be attributed to the economic conditions of the areas in which these hospitals are situated and the recruitment incentives they provide. However, it’s crucial to emphasize that having an education level of a bachelor’s degree or lower does not inherently determine a physician’s quality. The performance of a doctor is influenced by various factors, including individual abilities, specialized training, experience, and more. Notably, the immediate patient-doctor accessibility, as indicated by on-duty medical staff, is similar across both hospital types. However, a threefold difference in annual visits also indicates that county-level hospitals experience a significantly lower overall workload in their ERs compared to provincial or municipal hospitals. This lower patient volume can lead to a less intense work environment for the medical staff at county-level hospitals.

In our examination of the configuration of ED units and bed availability, we found that essential emergency care facilities, including emergency rooms, emergency observation rooms, and pre-hospital emergency care, are universally present, with a 100% representation in both county-level and provincial or municipal public hospitals. This reflects the widespread accessibility of crucial emergency care infrastructure. However, disparities emerge when considering the number of beds available in these facilities. County-level hospitals tend to have a slightly lower median number of beds in their emergency rooms, emergency observation rooms, and EICUs compared to their provincial or municipal counterparts. These variations in bed availability may influence patient capacity and have the potential to contribute to differences in the quality of care provided. It is important to note that despite these disparities, county-level hospitals continue to play a pivotal role in delivering emergency care, particularly in less urbanized regions. This underscores their significance in addressing the unique healthcare needs of these areas. Additionally, our data reveals that pre-hospital emergency care is well-supported in both settings, with an equal number of ambulances available. This suggests that access to pre-hospital care services remains consistent and is not significantly influenced by the type of hospital.

Regarding infrastructure, the higher bed availability in provincial or municipal hospitals echoes previous studies, which highlighted the superior funding and resources of urban hospitals. Nevertheless, our study suggests a potential for longer waiting times in county-level hospitals, an area that warrants further investigation. In addition, our findings regarding clinical skill capabilities are concerning, revealing that county-level hospitals may not be well-equipped to handle complex emergencies. This echoes Yan et al. (17) findings, which highlighted the uneven distribution of medical expertise across China. The lack of advanced clinical capabilities in county-level hospitals has serious implications for patient outcomes, especially in emergencies. Interestingly, while provincial or municipal hospitals manage a higher patient volume, they also have a superior CPR success rate, indicating not only better resources but also potentially more efficient resource utilization.

In addition, we observed that county-level hospitals frequently manage non-urgent cases, particularly those corresponding to Level IV in accordance with the “Expert Consensus on Emergency Pre-examination and Triage (11).” These non-urgent cases typically encompass minor injuries, minor illnesses, non-severe pain, or other medical conditions that do not involve an immediate threat to life. It’s important to emphasize that while these cases may not be life-threatening, they represent a substantial portion of the patient load in county-level hospitals. The ability of county-level hospitals to effectively manage non-urgent cases not only contributes to the overall efficiency of the healthcare system but also plays a vital role in relieving the burden on higher-level hospitals. This emphasizes the significance of these hospitals within the broader healthcare network.

Given the pivotal role of county-level hospitals, especially in less urbanized regions, there is a pressing need for targeted investments in these institutions. Efforts to enhance emergency care in county-level public hospitals should begin with a focus on infrastructure improvements. These hospitals must have the necessary facilities, equipment, and resources to handle emergencies effectively, including expanding bed capacity, equipping EDs with advanced medical equipment, and ensuring they are well-prepared to provide high-quality care. Addressing clinical skill gaps is equally vital. Prioritizing training and education for healthcare professionals in county-level hospitals can significantly enhance the capabilities of doctors and nurses. Continuous medical education and skill development programs should be implemented to keep these professionals up-to-date. One pressing concern is the low rate of ultrasound utilization, which requires immediate attention, and efforts should be concentrated on enhancing the availability and utilization of ultrasound equipment, as it holds the potential to markedly improve patient care and diagnostic accuracy.

Drawing from international best practices, there is potential to adopt telemedicine (18, 19), which has proven effective in rural areas of countries like the United States (20) and Australia (21). Telemedicine leverages the strides made in digital technology and telecommunications, enabling remote consultation, diagnosis, and even treatment. Incorporating telehealth into the emergency care framework of county-level hospitals is instrumental in extending the reach of specialized healthcare professionals to patients residing in less urbanized regions. This not only facilitates timely medical attention but also provides a mechanism to circumvent the resource limitations faced by these institutions. However, the successful integration of telehealth into emergency care is not without its challenges. Foremost among these challenges is the prerequisite of reliable and high-speed internet access, which may be deficient in certain rural areas. Tackling connectivity issues and establishing a robust telehealth infrastructure is imperative. Additionally, there exists a learning curve associated with adopting telemedicine practices among healthcare providers. This necessitates the provision of adequate training and support to ensure that medical professionals can effectively leverage telehealth tools.

However, this study has certain limitations. Its cross-sectional nature may not capture evolving improvements or declines in hospital capabilities. While we focused on Henan province, extrapolating these results to all of China might be ambitious, given the country’s vast geographical and developmental disparities. Additionally, the decision to include hospitals with over 500 beds may exclude smaller hospitals that also play crucial roles in the healthcare system. The exclusion of smaller hospitals represents a limitation in the scope of this study, as these hospitals may have unique characteristics and challenges. Furthermore, we did not directly address differences in funding among the hospital types in the survey. The primary reason for not doing so was to maintain the feasibility and simplicity of data collection, as funding can be a complex and multifaceted issue with various sources and allocation mechanisms. Finally, the study’s reliance on self-reported data raises concerns about potential biases, and while efforts were made to verify the data, there is the possibility of reporting inaccuracies.

## Conclusion

This study offers a comprehensive evaluation of EDs in county-level public hospitals in Henan, revealing significant disparities between county and provincial or municipal hospitals in staffing, infrastructure, clinical capabilities, and operational proficiency. The findings underscore the pressing need for targeted interventions and investments in county-level hospitals to improve the quality of emergency care. Policymakers and healthcare administrators must prioritize enhancing infrastructure, advancing clinical training, and adopting innovations like telemedicine to bridge the gap.

## Data availability statement

The original contributions presented in the study are included in the article/Supplementary material, further inquiries can be directed to the corresponding authors.

## Author contributions

YC: Conceptualization, Writing – original draft, Writing – review & editing. XC: Data curation, Methodology, Software, Writing – original draft, Writing – review & editing. JZ: Data curation, Formal analysis, Validation, Writing – original draft, Writing – review & editing. LX: Investigation, Software, Writing – review & editing. LQ: Funding acquisition, Writing – review & editing.
