# Supplementary material for: Comprehensive assessment of emergency departments in county-level public hospitals: a multicenter descriptive cross-sectional study in Henan province, China
Source: Front Public Health. 2023 Nov 14;11:1301030. doi: 10.3389/fpubh.2023.1301030 (PMC10682090; doi:10.3389/fpubh.2023.1301030)
Supplement: Supplementary file 1 [file Data_Sheet_1.docx]

**Section 1: General Information**

Hospital name:

City or country of location:

Does the hospital have a specialized emergency department?

Respondent's details:

Name:

Department:

Position:

Contact Information:

**Section 2: Emergency Department Human Resources**

Total number of doctors:

Number of male doctors:

Number of doctors aged 30-40:

Doctors educational background:

Master's degree or higher:

Bachelor's degree:

Below bachelor's degree:

Doctor titles:

Number of junior doctors:

Number of intermediate doctors:

Number of senior doctors:

Total number of nurses:

Number of male nurses:

Number of nurses aged 30-40:

Nurse educational background:

Master's degree or higher:

Bachelor's degree:

Below bachelor's degree:

Nurse titles:

Number of junior nurses:

Number of intermediate nurses:

Number of senior nurses:

Number of doctors on duty in the emergency room:

Number of nurses on duty in the emergency room:

**Section 3: Emergency Unit Setup**

Emergency Room:

Yes Number of beds:

No

Emergency observation room:

Yes Number of beds:

No

Emergency intensive care unit (EICU):

Yes Number of beds:

No

Pre-hospital emergency care:

Yes Number of ambulance:

No

**Section 4: Emergency Department Clinical Skill Capabilities**

Point-of-care testing (POCT), such as the bedside rapid blood gas testing, cardiac enzyme testing

Yes

No

Independent endotracheal intubation (emergency department physicians can independently perform without assistance from anesthesiologists)

Yes

No

Independent fiberoptic bronchoscopy (emergency department physicians can independently perform without assistance from pulmonologists)

Yes

No

Independent continuous renal replacement therapy (CRRT) (emergency department physicians can independently perform without assistance from nephrologists or blood purification centers)

Yes

No

Utilization of bedside ultrasound equipment

Yes

No

Independent ECMO techniques

Yes

No

Intraosseous (IO) infusion techniques:

Yes

No

**Section 5: Emergency Room Basic Operational Capabilities**

Number of patients treated annually:

Severity level

Level 1: Critical patients:

Level 2: Severe patients:

Level 3: Urgent patients:

Level 4: Non-urgent patients:

Annual patient deaths:

Annual pre-hospital cardiopulmonary resuscitation (CPR) cases:

Cases of successful return of spontaneous circulation (ROSC) (defined as patients with a persistent circulation for at least 20 consecutive minutes not requiring chest compressions)

Annual in-hospital CPR cases:

Cases of successful ROSC:
